# Fevers in Alabama

**Published:** 1849-01

**Authors:** 


### FEVERS IN ALABAMA.

In a letter dated Nov. 5th, 1848, Dr. T. C. Osborne, speaking of the prevalence of disease in Alabama during the past season, mentions that on "one plantation in the neighborhood of Erie two hundred and seventy-five cases of fever had occurred, of which two were cases of typhus, twenty-five of typhoid, and the remaining two hundred and forty-eight cases of remittent and intermittent fever. Only five of the whole proved fatal. One sank under copious hemorrhage from the bowels; one died without any apparent lesion; and two died in convulsions. The boy who was carried off by hemorrhage, had been for sometime convalescent from typhoid fever. The girl, in whose case no special lesion was apparent, presented in her disease all the symptoms of typhus."

